# The Association between Low Body Weight and Scoliosis among Korean Elementary School Students

**DOI:** 10.3390/ijerph15122613

**Published:** 2018-11-22

**Authors:** Kyoungkyu Jeon, Dong-il Kim

**Affiliations:** 1 Department of Sports Science, Incheon National University, Incheon 22012, Korea; jeonkay@inu.ac.kr; 2 Sport Science Institute, Incheon National University, Incheon 22012, Korea; 3 Department of Professional Therapy, Graduate School of Professional Therapy, Gachon University, Gyeonggi-do 13120, Korea

**Keywords:** Korean elementary school students, scoliosis, body composition, low body weight

## Abstract

*Background:* The prevalence of scoliosis in Korean elementary school students is increasing, leading to various physical and psychological problems. This study aimed to investigate the association between low body weight and scoliosis among Korean elementary school students. *Methods:* This was a cross-sectional analysis. Participants were 1062 elementary school students in the metropolitan areas of Korea. Participants were evaluated for scoliosis based on body composition, including weight and height, and with spine structure analysis equipment. Scoliosis diagnosis was defined as having a Cobb’s angle greater than 10°. *Results:* Participants were divided into Normal Weight (NW), Underweight (UW), and Severely Underweight (SUW) groups. Results show that the UW and SUW groups had significantly higher risks of developing scoliosis (odds ratio (OR): 1.43, 95% CI (confidence interval): 1.07–1.90; OR: 1.45, 95% CI: 1.02–2.05) compared to the NW group; after controlling for age and gender, the OR were 1.44 (95% CI: 1.08–1.92) and 1.46 (95% CI: 1.01–2.09), respectively. *Conclusions:* Low weight and the risk of developing scoliosis are very closely associated. Maintenance of appropriate and normal weight in Korean elementary school students appears to be a very effective method for preventing and reducing the risk of scoliosis.

## 1. Introduction

The prevalence of scoliosis among Korean elementary school students aged 10 to 14 years old has increased approximately 4.5 times, from 1.35% in 2002 (male: 0.95%, female: 1.75%) to 6.17% in 2008 (male: 3.9%, female: 8.59%), and continues to increase [1]. The type of scoliosis known to occur in children and adolescents is, by nature, structural scoliosis, which accounts for more than 80% of all cases, with unclear etiology [2,3]. Scoliosis typically develops in late childhood, near the age of 10 years old, or early adolescence; this represents a severe public health problem, with more than 4% of adolescents between the ages of 11 to 17 years old showing spinal malformation [2]. The occurrence of scoliosis is typically attributed to posture instability (which could be due to prolonged computer use and desks unsuitable for children’s physiques), and developmental destabilization, all of which are environmental factors associated with scoliosis [4,5].

Scoliosis is typically diagnosed when the Cobb’s angle is greater than 10°, as identified through spinal x-rays, and is treated using methods such as exercise therapy, braces, and surgery, depending on the age of the patient and the severity of symptoms [3,6]. Scoliosis involves the gradual change of the body physique, including rachiocampsis; such changes to the body can negatively influence the formation of body image during childhood and adolescence, when there is a high level of interest in outer appearance [7]; it may also lead to a complex set of problems including disorders of the musculoskeletal systems, as well as psychological and social problems [6].

Studies conducted overseas have reported that low weight contributes strongly to the occurrence of scoliosis [8,9]. However, the problem of low weight among Korean children has been overlooked due to the severity of problems related to overweight and obesity. In particular, there are a lack of studies on the association between low body weight and scoliosis in Korean children. Furthermore, low weight has a negative influence on physical and mental health [10,11], and these problems are especially pronounced for children as they experience rapid growth in their muscular and skeletal systems, during the second phase of growth. As such, maintenance of an appropriate weight level is very important for reducing the risk of scoliosis [11,12].

There have been active attempts to identify factors that influence mental and social disorders among children diagnosed with scoliosis, as well as attempts to develop effective intervention methods to reduce changes to the spinal angle outside of Korea [13]; however, there are very few studies on scoliosis that focus on children in Korea.

The purpose of our study was to examine the association between low body weight and scoliosis among Korean elementary school students, thereby providing basic data on exercise and lifestyle modifications to improve growth and development of children with scoliosis.

## 2. Materials and Methods

### 2.1. Participants

This study was a cross-sectional analysis that randomly sampled three elementary schools located within the metropolitan area of Korea, with the principals and teachers having understood the research background and purpose, and having agreed to participate in the study. A total of 82 classes are within the three elementary schools that participated in this study and the total number of students initially enrolled was 1062 students. All elementary school students participating in this study understood the purpose of the study, and their respective guardians signed the participation agreement form. Among the total group of students, 97 students who had not been administered tests evaluating body composition or scoliosis were excluded; thus the final study population was 965 students in 2017 years. All participants provided written informed consent, and the study was approved by the Institutional Review Boards at Incheon National University [IRB (No. 7007971-201612-003-01)]. The physical characteristics of the participants of are shown in Table 1.

### 2.2. Study Tools

#### 2.2.1. Body Measurements

Body measurements were conducted by nurses and trained research staff. Participants were dressed in comfortable clothing before measuring their height, weight, and body composition using an extensometer (Seca, Germany) and body composition analytic equipment (Inbody 720, Biospace, Korea).

#### 2.2.2. Identifying Scoliosis

**Figure 1:**
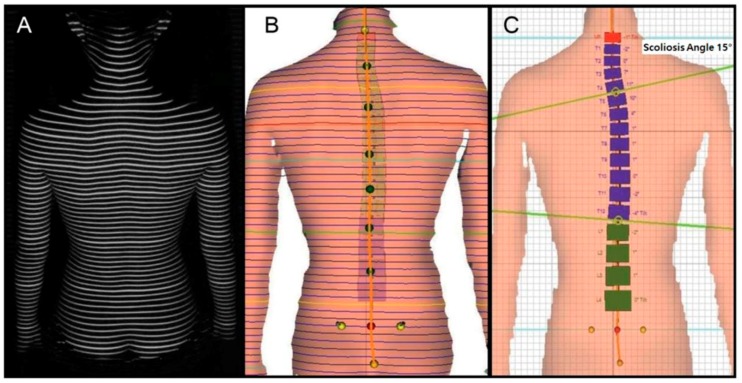
Measurement by rasterstereography image of patient’s surface back in upright position. (**A**) The back of the trunk of the subject is photographed through a halogen light source; (**B**) Reference setting of rasterstereo-line for back of trunk; (**C**) Reconstruction into a three-dimensional model.

To measure the spinal deformations of study participants, we applied spinal structure analysis equipment (Formetric 4D, Dires International GmbH, Schlangenbad, Germany) that uses the rastersterography method to aim a halogen light source on the surface behind the participants’ back, obtaining a video using triangulation and recreating the results into a three-dimensional model. The equipment displays an average deviation of less than 0.15 mm in the video capturing the surface behind the trunk, and an accuracy of average deviation through the lateral deviation within 3 degrees [14,15]; as such, it is used to diagnose spinal deviations. This equipment has the benefit of short measurement times, between approximately 0.04–6 s, and the participant is sitting in an upright position. The asymmetrical form of the pelvis and spinal deviations as well as the spinal structure are presented relatively accurately with the error of the three-dimensional spinal model being within 0.05 mm. As such, it is possible to predict the effects of rehabilitative exercise intervention and treatment. During measurement, the anatomical apex of the C7 vertebral body process, sacrum, and left and right posterior superior iliac spine (PSIS) of the participant are used as the bases for analyzing the clip. Participants were asked to remove their top clothing to reveal the PSIS to ensure that the halogen light source could form the rasterstereo-line on all vertebral processes, including the C7 spinal process, behind the trunk (Figure 1). As this line can help visualize accurate spinal structure in a clip, the space used for measurement was darkened in order to maximize the penetration of the halogen light source on the back side of the participants trunk. Based on these methods, we measured the length of the trunk (length, mm), its inclination (mm), imbalance (mm), and torsion (°), the inclination (°), rotation (°) of the pelvis, and the scoliosis angle (°). Per the above analysis method, this study’s diagnostic definition of scoliosis was defined as having a Cobb’s angle greater than 10°.

### 2.3. Statistical Analysis

Statistical analysis was performed using SPSS/Window 23.0 (SPSS Inc., Chicago, IL, USA). This study used independent *t*-tests to comparatively analyze the average values of measurement items between groups according to gender, divided according to body mass index (BMI) into 3 groups: Normal Weigh (NW) (18.5 ≤ BMI < 25), Underweight (UW) (16 ≤ BMI < 18.5), and severely Underweight (SUW) groups. These groups were used to investigate risk factors of scoliosis, and comparatively analyze body composition and scoliosis risk factors using analysis of covariance (ANCOVA) while controlling for age and gender. Lastly, we compared the odds ratio (OR) of scoliosis prevalence for NW, UW, and SUW groups through logistic regressions after controlling for age and gender. The statistical significance level was set at *p* < 0.05.

## 3. Results

### 3.1. Correlation between BMI, Body Composition, and Scoliosis Risk Factors

Table 2 reports scoliosis risk factors according to BMI. The UW and SUW groups had significantly lower levels of lean mass, percent body fat, trunk length, and trunk inclination, but had significantly higher scoliosis angles (NW: 10.18 ± 3.79°, UW: 11.06 ± 4.29°, SUW: 11.26 ± 4.71°).

### 3.2. Correlation between BMI and Risk of Scoliosis

Table 3 reports the risk of scoliosis development according to BMI. Compared to the NW group, the UW and SUW groups had significantly higher risks of developing scoliosis (OR: 1.43, 95% confidence interval (CI): 1.07–1.90 for the UW group and OR: 1.45, 95% CI: 1.02–2.05) for the SUW group, respectively). After controlling for age and gender, the risk of scoliosis was still significant (OR: 1.44, 95% CI: 1.08–1.92 and OR: 1.46, 95% CI: 1.01–2.09, respectively).

## 4. Discussion

The purpose of this study was to investigate the association between low body weight and scoliosis among Korean elementary school students. In overseas countries, the government is directly involved in testing for scoliosis among school children; these tests have been widely distributed over the last 20 years, and are currently conducted in many countries, including the United States, Sweden, South Africa, and Japan [16,17,18,19]. The Scoliosis Research Society recommends yearly testing for all children between the ages of 10 to 14 years; such early diagnosis of scoliosis among elementary school students prevents spinal deformation and identifies symptoms at an early stage, leading to improved prognoses [20,21]. However, Korea does not provide scoliosis testing for elementary school students.

It has been reported that scoliosis correlates closely with gender, age, obesity, curve of the spine, and the curvature direction [8,9,22]. Moreover, it has been reported that girls are at higher risk of scoliosis compared to boys [23,24]. Wong et al. (2005) reported that the prevalence of scoliosis among boys aged 11 to 12 years old and aged 13 to 14 years old were 0.21% and 0.66%, respectively; the figures for girls of the same ages were 1.37% and 2.22%, respectively, indicating that girls have a higher prevalence of scoliosis compared to boys [25]. This current study also showed significant differences in trunk torsion between the male and female groups (male: −0.38 ± 5.94, female: 0.34 ± 5.50, *p* = 0.047); while not statistically significant, there were also differences in the scoliosis angle between groups (male: 10.52 ± 4.07, female: 10.98 ± 4.35, *p* = 0.088).

Scoliosis was found to correlate with gender and age [22]; in particular, it was associated with low weight and eating disorders (anorexia) [8]. In the study by Catherine et al. (2003) including 905 female adolescents [598 adolescent idiopathic scoliosis (AIS) girl, 307 healthy girl], the AIS group showed a significantly lower BMI and weight compared to the healthy group [9]. Moreover, in a study by Yong et al. (2009) including 93,626 children aged between 9 to 13 years old, the low weight group had a 1.5 times higher risk of scoliosis compared to the healthy and overweight groups (OR: 1.5, 95% CI: 1.2–1.8) [12]. The current study also indicated that the UW and SUW groups had significantly higher risks of developing scoliosis, having risk levels that were 1.43 times (95% CI: 1.07–1.90) and 1.45 times (95% CI: 1.02–2.05) higher, respectively; after controlling for age and gender, these figures remained significant (OR: 1.44, 95% CI: 1.08–1.92 and OR: 1.46, 95% CI: 1.01–2.09, respectively; Table 3). The UW and SUW groups had significantly lower levels of muscular mass and percent body fat (Table 2). These results indicate that lower BMI leads to lower muscle mass, producing negative effects on the stability of the muscular and skeletal systems (spine-related) and increased risk of scoliosis. Based on the above data, low weight is very closely linked with scoliosis [8,9]. Low weight leads to a reduction in bone density, and this reduced bone density can have severe negative effects on patients with scoliosis, such as changes to their skeletal structure [26]. As such, low weight increases the risk of scoliosis and has an extremely negative influence on body composition, including bone density, increasing the risk of various complications.

This study has several limitations. First, participants of this study are elementary school students, and the data therefore cannot be extrapolated to individuals of different age ranges. Second, this study observed associations between the risk of scoliosis and level of obesity, rather than a cause-and-effect relationship; thus the results of this study cannot be extrapolated as such. Existing studies published overseas on children with scoliosis have actively focused on the causes, diagnosis, and treatment of scoliosis [4,27], but domestic research remains very limited. Specifically, there are no studies evaluating the risk of scoliosis associated with low weight using a sufficient sample size, and this study appears to be valuable as the first study of this kind.

## 5. Conclusions

Our results suggest that among Korean elementary school students, low weight is associated with scoliosis. By using a variety of statistical analyses, our study emphasizes the association and importance of low weight with the prevalence of scoliosis.

## Figures and Tables

**Table 1:** Subject characteristics.

Variables	Male (*n* = 504)	Female (*n* = 461)	*p*-Value
Age (years)	11.15 ± 1.21	11.14 ± 1.22	0.985
Weight (kg)	38.82 ± 8.99	38.17 ± 17.94	0.467
Height (cm)	143.97 ± 9.86	143.80 ± 11.32	0.810
Lean mass (kg)	28.13 ± 6.12	26.17 ± 5.24	<0.001
Percent body fat (%)	22.38 ± 7.94	24.87 ± 7.11	<0.001
BMI (kg/m^2^)	18.51 ± 2.59	17.85 ± 2.50	<0.001
Trunk Length (mm)	369.39 ± 34.69	375.79 ± 36.80	0.006
Trunk Inclination (mm)	13.42 ± 19.75	12.71 ± 18.43	0.565
Trunk Imbalance (mm)	−2.07 ± 8.86	−1.42 ± 9.40	0.265
Trunk Torsion (°)	−0.38 ± 5.94	0.34 ± 5.50	0.047
Pelvic Inclination (°)	0.18 ± 2.85	0.23 ± 2.72	0.812
Pelvic Torsion (°)	0.78 ± 2.46	0.87 ± 2.18	0.535
Pelvic Rotation (°)	−1.32 ± 4.23	−0.91 ± 4.63	0.148
Scoliosis Angle (°)	10.52 ± 4.07	10.98 ± 4.35	0.088

Date and abbreviation are mean ± standard deviation; Abbreviations: BMI, body mass index.

**Table 2:** Variables according to body mass index (BMI).

Total (*n* = 965)	BMI		
	1st (*n* = 391) Normal Weight (18.5 ≤ BMI < 25)	2nd (*n* = 375) Underweight (16 ≤ BMI < 18.5)	3rd (*n* = 199) Severely Underweight (BMI < 16)
Multivariable-adjusted ^Ұ^			
Lean mass (kg)	30.21 ± 5.48	26.69 ± 5.20 *	22.19 ± 3.12 * ^
Percent body fat (%)	29.08 ± 6.67	20.77 ± 6.08 *	18.01 ± 4.68 * ^
Trunk Length (mm)	380.72 ± 35.08	371.27 ± 37.28 *	358.43 ± 29.47 *
Trunk Inclination (mm)	16.70 ± 19.09	12.31 ± 18.74 *	7.43 ± 18.45 * ^
Trunk Imbalance (mm)	−1.57 ± 9.36	−2.00 ± 8.93	−1.68 ± 9.03
Trunk Torsion (°)	−0.24 ± 5.37	−0.09 ± 5.59	0.47 ± 6.68
Pelvic Inclination (°)	0.05 ± 2.73	0.35 ± 2.72	0.24 ± 3.01
Pelvic Torsion (°)	0.48 ± 2.41	0.98 ± 2.24 *	1.21 ± 2.26 *
Pelvic Rotation (°)	−1.03 ± 4.20	−1.28 ± 4.45	−1.02 ± 4.83
Scoliosis Angle (°)	10.18 ± 3.79	11.06 ± 4.29 *	11.26 ± 4.71 *

Data are mean ± standard deviation. Abbreviations: BMI, body mass index. * significant with 1st group, ^ significant with 2nd group, group *p* < 0.05. ^Ұ^ Adjusted for age and gender.

**Table 3:** Odds ratio according to BMI.

Total (*n* = 965)	BMI		
	1st (*n* = 391) Normal Weight (18.5 ≤ BMI < 25)	2nd (*n* = 375) Underweight (16 ≤ BMI < 18.5)	3rd (*n* = 199) Severely Underweight (BMI < 16)
OR (95% CI)			
**Scoliosis**	1	1.43 (1.07–1.90)	1.45 (1.02–2.05)
Age-adjusted OR (95% CI)			
**Scoliosis**	1	1.44 (1.08–1.92)	1.48 (1.04–2.12)
Age and gender-adjusted OR (95% CI)			
**Scoliosis**	1	1.44 (1.08–1.92)	1.46 (1.01–2.09)

Abbreviations: CI, confidence interval; OR, Odds ratio; BMI, body mass index.
